# Analysis of the Transcriptome in Hyperoxic Lung Injury and Sex-Specific Alterations in Gene Expression

**DOI:** 10.1371/journal.pone.0101581

**Published:** 2014-07-08

**Authors:** Krithika Lingappan, Chandra Srinivasan, Weiwu Jiang, Lihua Wang, Xanthi I. Couroucli, Bhagavatula Moorthy

**Affiliations:** 1 Department of Pediatrics, Section of Neonatology, Texas Children's Hospital, Baylor College of Medicine, Houston, Texas, United States of America; 2 Division of Pediatric Cardiology, Department of Pediatrics, University of Texas Medical School at Houston, Houston, Texas, United States of America; University of Texas Medical Branch, United States of America

## Abstract

Exposure to high concentration of oxygen (hyperoxia) leads to lung injury in experimental animal models and plays a role in the pathogenesis of diseases such as Acute Respiratory Distress Syndrome (ARDS) and Bronchopulmonary dysplasia (BPD) in humans. The mechanisms responsible for sex differences in the susceptibility towards hyperoxic lung injury remain largely unknown. The major goal of this study was to characterize the changes in the pulmonary transcriptome following hyperoxia exposure and further elucidate the sex-specific changes. Male and female (8–10 wk) wild type (WT) (C57BL/6J) mice were exposed to hyperoxia (FiO_2_>0.95) and gene expression in lung tissues was studied at 48 h. A combination of fold change ≥1.4 and false discovery rate (FDR)<5% was used to define differentially expressed genes (DEGs). Overrepresentation of gene ontology terms representing biological processes and signaling pathway impact analysis (SPIA) was performed. Comparison of DEG profiles identified 327 genes unique to females, 585 unique to males and 1882 common genes. The major new findings of this study are the identification of new candidate genes of interest and the sex-specific transcriptomic changes in hyperoxic lung injury. We also identified DEGs involved in signaling pathways like MAP kinase and NF-kappa B which may explain the differences in sex-specific susceptibility to hyperoxic lung injury. These findings highlight changes in the pulmonary transcriptome and sex-specific differences in hyperoxic lung injury, and suggest new pathways, whose components could serve as sex-specific biomarkers and possible therapeutic targets for acute lung injury (ALI)/acute respiratory distress (ARDS) in humans.

## Introduction

Exposure to high concentration of oxygen (hyperoxia) leads to lung injury in experimental animal models and plays a major role in the pathogenesis of diseases such as Acute Respiratory Distress Syndrome (ARDS) and Bronchopulmonary dysplasia (BPD) in humans [1]–[3]. Reactive oxygen species (ROS) generated during hyperoxia exposure result in damage of many cellular components including DNA, proteins and lipids and ultimately leading to epithelial and endothelial cell death in the lung. Hyperoxia is also associated with increased expression of cytokines that sequester and activate inflammatory cells, most notably, neutrophils [4], [5]. Together these events lead to pulmonary edema and atelectasis, which are the key histopathological findings in acute lung injury [6]. Hyperoxia exposure leads to pathological changes similar to ARDS in other mammalian species [1]–[5], [7], [8].

Sex-specific differences in lung injury have been established both in animal models of acute lung injury (ALI) and observed in epidemiological studies in human patients. Males with ARDS have a higher mortality rate compared to females [9]–[11]. Sex-based differences have also been observed in diseases such as idiopathic pulmonary fibrosis and asthma [12]–[15]. In premature neonates, male sex is considered to be an independent risk factor for the development of BPD [12], [16]–[18]. The reasons behind these sex-biased differences in lung injury are not well elucidated yet. Differences in lung development among male and female fetuses may contribute to these differences. Hormonal differences between males and females are also thought to play a role [19], [20]. Estrogen is known to have a stimulatory and androgens an inhibitory effect on lung development [19]. Expression of enzymes involved in antioxidant defense pathways have also been shown to be different between male and female premature neonates [21], [22]. In acute lung injury models, testosterone was found to increase [23] and estrogen to ameliorate inflammation and injury [24]. Studies on sex hormones have identified some potential mechanisms behind the sexually dimorphic differences but more work is needed.

Like many other diseases genetic and non-genetic factors play a role in hyperoxic lung injury. Genetic susceptibility is thought to contribute to the development of BPD in human neonates [25]. Some mouse strains are resistant (C3, 129X1/SvJ), while others are susceptible (B6, A/J and D2) to hyperoxic lung injury [26]. *Nrf2*-ARE (antioxidant response element) signaling pathway has been identified as significant in many acute lung injury models [27]. QTL (quantitative trait loci) analyses has revealed other candidate genes; angiopoietin-1 (*Angpt1*) and oxidation resistance-1 (*Oxr1*) which may determine susceptibility to hyperoxic lung injury [28].

We have recently shown that after hyperoxia exposure, adult male mice show greater pulmonary edema, lung injury, inflammation and cell death compared to female mice [29]. However, the molecular mechanisms of sex differences are not completely understood. Sex-specific global gene expression in an animal model of hyperoxic lung injury has not been reported. In this study, we employed a non-biased approach to measure global changes in gene expression following hyperoxia exposure in the lung at 48 h and further studied the sex-specific changes in male and female mice.

## Materials and Methods

### Animals

This study was conducted in strict accordance with the recommendations in the Guide for the Care and use of Laboratory Animals of the National Institutes of Heath. The Institutional Animal Care and Use Committee (IACUC) of Baylor College of Medicine approved the protocol (Protocol number AN-907). All efforts were made to minimize suffering. Breeding pairs of mice were obtained from the Jackson Laboratory (Bar Harbor, ME). 8–10 week old male (C57BL/6J) or female mice were maintained at Texas Children's Hospital animal facility and used for the study. They were fed standard mice food and water *ad libitum*. Animals were maintained in a 12 h day/night cycle.

### Oxygen exposure

Mice were maintained in either room air (21% oxygen) or exposed to hyperoxia (95–100% oxygen) environment using pure O_2_ at 5 l/min for 24–72 h in a sealed Plexiglass chamber, as reported previously [30]. After sealing the chamber, the oxygen concentration in the plexiglass chamber was measured frequently by an analyzer (Getronics, Kenilworth, New Jersey). Purified tap water and food (Purina Rodent Lab Chow 5001 from Purina Mills, Inc., Richmond, IN) were available *ad libitum*. After hyperoxia exposure, the animals were anesthetized with 200 mg/kg of sodium pentobarbital (i.p.) and euthanized by exsanguination while under deep pentobarbital anesthesia. The lung tissues were harvested for further analysis.

### RNA isolation

We used a total of 12 animals per sex per treatment group. Total RNA from lung samples in mice exposed to room air or hyperoxia for 48 h was isolated using the miRNeasy kit as per the manufacturer's standard protocols (Qiagen, Valencia, CA, USA). Following total RNA isolation, sample concentration was assayed using a Nanodrop-8000 (Thermo Scientific, Wilmington, DE, USA). Sample Quality checks were done using the NanoDrop spectrophotometer and Agilent Bioanalyzer.

### Affymetrix Gene Chip Array Analysis

We used the Ambion (Life Technologies, Grand Island, NY, USA) WT Expression Kit protocol and reagents to convert total RNA into sense-strand cDNA. The cDNA was then fragmented and labeled using the Affymetrix GeneChip (Affymetrix, Santa Clara,CA,USA) WT Terminal Labeling Kit.

### Data analysis

We used 3 biological replicates in each group. The groups were: 1) Room air-male, 2) Room air-female, 3) Hyperoxia-male and 4) Hyperoxia-female (NCBI GEO accession number: GSE51039). To minimize the impact of individual mouse to mouse variability on the microarray data, each biological replicate was comprised of a randomized pool of lung RNA prepared from n = 4 mice. Gene expression in lung tissue was studied using the Mouse Gene 1.0 ST Array (Affymetrix). Raw gene expression data was preprocessed using ‘Robust Multichip Average’ methodology. A combination of fold-change (FC)≥1.4 and false discovery rate (FDR)<5% was used to define differentially expressed genes (DEGs). Overrepresentation of gene ontology terms representing biological processes among the DEGs was tested using a conditional hypergeometric test (p-value <0.01). Signaling pathway impact analysis (SPIA), was also performed, as this approach combines two types of evidence: (i) the over- representation of DEGs in a given pathway and (ii) the abnormal perturbation of that pathway, as measured by propagating measured expression changes across the pathway topology [31]. A significance threshold of 5% was used on the FDR corrected p-values in order to detect pathway significance.

### Real time qPCR validation

To validate microarray results, a subset of genes was validated by quantitative real-time PCR (qRT-PCR). Non-random sampling strategies have limited utility as gene selection procedures because the validation results may not generalize to the entire set of DEGs [32]. Therefore, we selected 5 genes (*Ankrd1*, *Slc7a5*, *Slc7a11*, *Egr1* and *Nqo1*) from the pool of DEGs in a random-stratified manner. For random- stratified sampling, the entire list of upregulated genes was rank ordered according to fold change; the data were then divided into 5 equal-sized bins and one gene per bin was selected randomly. In addition, we also selected five additional genes from the pool of genes showing sex-specific differential expression in the microarray experiment. RNA (50 ng), isolated as above, was subjected to one step real time quantitative TaqMan RT-PCR using 7900HT Fast Real-Time PCR System (Applied Biosystems, Foster City, CA). Gene-specific primers purchased from life science technologies (Table 1) in the presence of TaqMan reverse transcription reagents and RT reaction mix (Applied Biosystems, Foster City, CA) were used to reverse transcribe RNA, and TaqMan Gene Expression probes and TaqMan Universal PCR Master Mix (Applied Biosystems, Foster City, CA), were used for PCR amplification. 18S was used as the reference gene. Concordance correlation coefficient (CCC) was used to measure the agreement between the log_2_ FC results from microarray and FC from qRT-PCR in both males and females. Data was processed and analyzed using Bioconductor software packages (*‘oligo’, ‘limma’, ‘GOstats’, ‘SPIA’, ‘HTqPCR’*) in R programming language [33], [34].

**Table 1:** List of genes selected for validation by qPCR analysis.

Gene symbol	Assay ID
Slc7a11	Mm00442530_m1
Ankrd1	Mm00496512_m1
Slc7a5	Mm00441516_m1
Nqo1	Mm01253561_m1
Egr1	Mm00656724_m1
BDNF	Mm04230607_s1
Pdk4	Mm01166879_m1
Agr2	Mm01291804_m1
IL1b	Mm00434228_m1
18s	Mm03928990_g1

### Histopathology, immunohistochemistry and western blot

The mice were maintained in either room air (21% oxygen) or exposed to hyperoxia (95–100% oxygen) environment using pure O_2_ at 5 l/min for 24 h, 48 h or 72 h in a sealed Plexiglass chamber, as described previously. Tracheotomy was performed on the anesthetized mice (n = 5/group) and the lung tissue was fixed by intratracheal instillation of 10% zinc formalin at constant pressure of 25 cm of H_2_O. Samples were left in solution for 24 h in formaldehyde, and then transferred to 70% ethanol for long-term storage. Routine histology was performed on lung tissues from individual animals following staining of the paraffin sections with hematoxylin and eosin. Five microns deparaffinized lung sections were immunostained with Anti xCT antibody (ab37186; dilution 1∶1000) followed by staining with biotinylated secondary antibodies (Vector Laboratories Burlingame, CA). For western blotting, lung whole protein (20 µg of protein) from individual male animals (n = 5/group) exposed to room air or to hyperoxia for 48 h, was prepared in Milliplex MAP lysis buffer (EMD Millipore) and used for apoprotein estimation. The primary antibody was the same as used in immunohistochemistry at a dilution of 1∶3000. For loading controls, the membranes were stripped and incubated with antibodies against β-actin, followed by electrochemical detection of bands. The statistical analysis of densitometric values was done using Students t-test and p value<0.05 was considered significant.

## Results

### Lung histopathology after hyperoxia exposure and sex-specific differences

**Figure 1:**
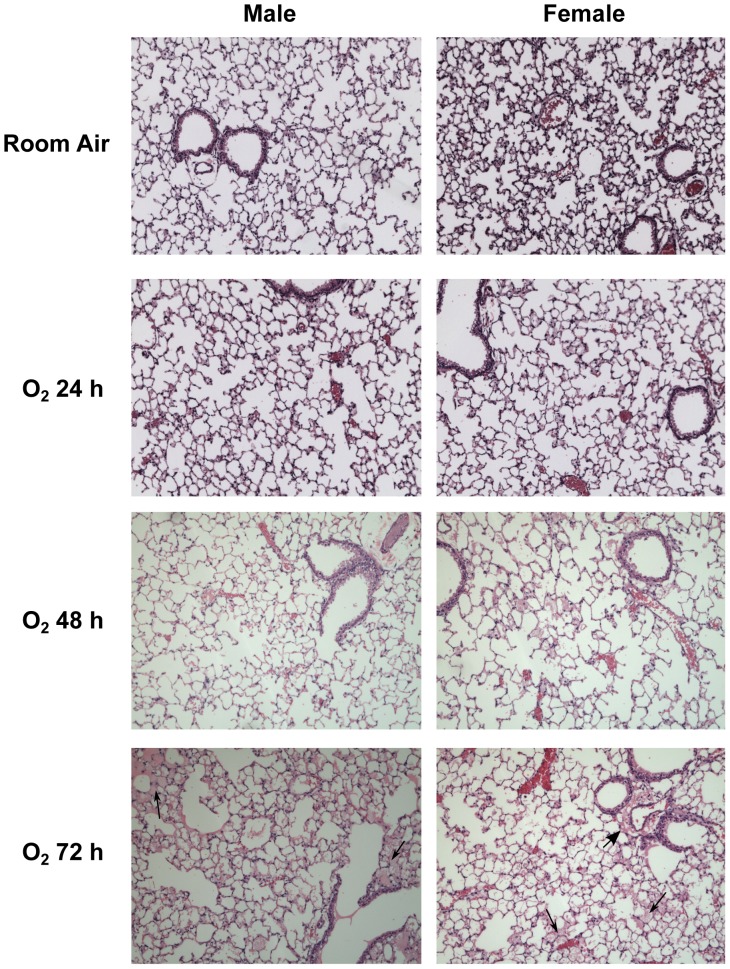
Representative hematoxylin eosin stained images from the lungs of male and female mice (n = 5 mice per group) exposed to room air or 24–72 h of hyperoxia. WT males show more alveolar edema and hemorrhage when compared to female mice. Thin arrows point to areas of alveolar edema. Thick arrow points to area of perivascular edema.

Figure 1 shows representative lung fields at 10× magnification from male and female mice exposed to room air or 24 h, 48 h or 72 h of hyperoxia. Qualitative assessment of lung injury showed that at 24 h there were no appreciable signs of lung injury due to hyperoxia exposure on histopathology. After 48 h of hyperoxia exposure, there are signs of lung injury in the form of alveolar edema. After 72 h, extensive lung injury in the form of alveolar, perivascular and peribronchial edema and alveolar hemorrhage was seen. Male mice showed more injury compared to female mice.

### Hyperoxia and sex-biased gene expression

**Figure 2:**
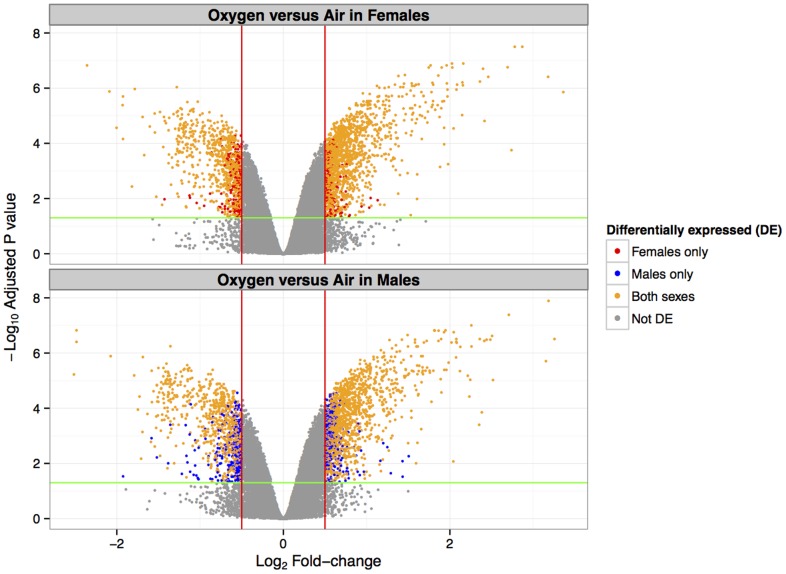
Volcano plot for differentially expressed genes in male and female mice after hyperoxia (FiO_2_>95%) exposure for 48 h compared to room air controls. The x-axis is log_2_fold change. Each point represents an individual transcript. The vertical lines represents 1.4 fold change with upregulated genes on the right and downregulated genes on the left. The horizontal lines represent an adjusted p value of 0.05.

**Figure 3:**
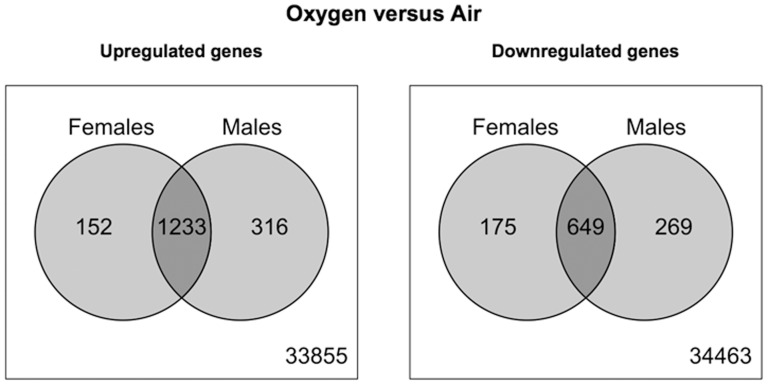
Venn diagram showing the comparison of upregulated and downregulated DEGs in the lung between male and female mice after hyperoxia exposure (FiO_2_>95%) for 48 hours.

After 48 h of hyperoxia exposure, a total of 2209 (upregulated (UR): 1385, downregulated (DR): 824) and 2467 (UR: 1549, DR: 918) genes were differentially expressed compared to room-air in females and males, respectively. Comparison of DEG profiles identified 327 genes unique to females, 585 unique to males, and 1882 common genes. Figure 2 shows the volcano plot of the DEGs (upregulated and downregulated) in males and females only and those that were differentially regulated irrespective of sex. Figure 3 shows the number of genes up and downregulated after hyperoxia exposure and the distribution in males and females, and those that were common to both male and female mice. Interestingly, 152 genes were upregulated in females only and 316 in males only. Among the downregulated genes, 175 were in females and 269 in males only as shown in Figure 3. Table 2 shows the genes with the highest fold change (≥4) in both males and females and (≥2) in males and females separately.

**Table 2:** List of pulmonary DEGs after hyperoxia exposure.

Common in both males and females (≥4 fold change)			
Affymetrix ID	Symbol	Chromosome	log_2_FC
10579331	Gdf15	chr8	3.36
10498024	Slc7a11	chr3	3.18
10534667	Serpine1	chr5	2.87
10545672	Mthfd2	chr6	2.78
10467191	Ankrd1	chr19	2.74
10361091	Atf3	chr1	2.69
10495675	F3	chr3	2.46
10598976	Timp1	chrX	2.42
10523182	Areg	chr5	2.40
10424400	Myc	chr15	2.36
10543067	Asns	chr6	2.16
10556297	Adm	chr7	2.16
10537146	Akr1b8	chr6	2.15
10513739	Tnc	chr4	2.15
10350516	Ptgs2	chr1	2.09
10448307	Tnfrsf12a	chr17	2.08
10467136	Ch25h	chr19	2.05
10582275	Slc7a5	chr8	2.03
10545130	Gadd45a	chr6	2.03
**Males only (≥2 fold change)**			
10543017	Pdk4	chr6	1.50
10507908	Fhl3	chr4	1.29
10587323	Gsta1	chr9	1.25
10517067	Sfn	chr4	1.17
**Females only (≥2 fold change)**			
10574427	Impdh2	chr8	1.03

### Pathway analysis of DEGs

To identify the biological processes that were enriched in the subsets of DEGs that were common to males and females, males only, and females only, we performed both over-representation and pathway topology analyses in these subsets of genes to highlight the differences in the results obtained by these two methods. There are many drawbacks to the over-representation analysis method (ORA) for pathway analysis. ORA typically only considers the number of genes and ignores any values associated with them such as probe intensities; it typically uses only the most significant genes and discards the others. By treating each gene equally, ORA assumes that each gene is independent of the other genes and that each pathway is independent of other pathways [35]. To overcome these drawbacks, we also performed pathway topology analysis. This type of analysis considers the structure and dynamics of an entire pathway by incorporating a number of important biological factors, including changes in gene expression, types of interactions, and the positions of genes in a pathway [31].

**Figure 4:**
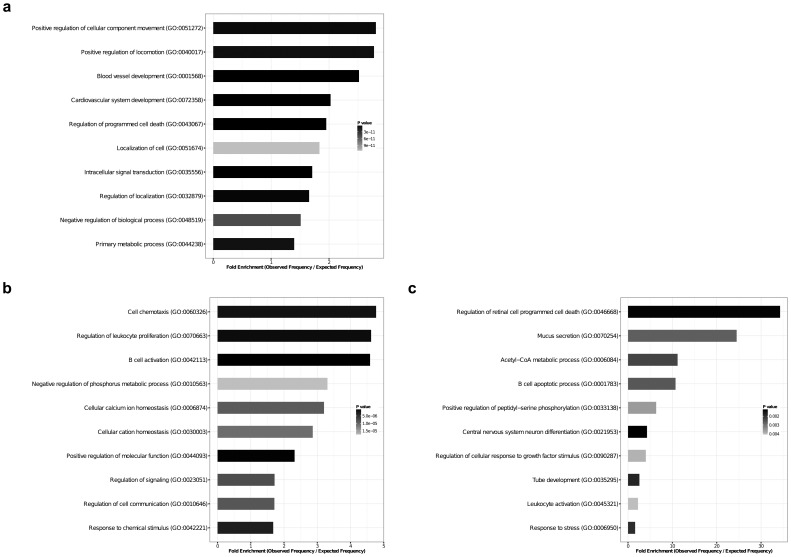
Pathway analysis by ORA (over representation analysis). Analysis of enrichment of biological processes in the three groups; 4a: analysis of DEGs common in both male and female animals after hyperoxia exposure 4b: analysis of DEGs in male animals 4c: analysis of DEGs in female animals. Overrepresentation of gene ontology terms representing biological processes among the DEGs was tested using a conditional hypergeometric test (p-value <0.01).

Figure 4a shows the major biological processes for the group of genes that were differentially regulated both in males and females. Among these were regulation of programmed cell death and angiogenesis. We then identified the enriched biological processes in DEGs, which showed sex-specific expression upon exposure to hyperoxia. Figure 4b shows the biological processes that were found to be significant among the DEGs in males only, and figure 4c shows the biological pathways that were found to be significant in females only. In males, the molecular pathways regulating leukocyte proliferation, adhesion, and chemotaxis were significant. In females, acetyl-CoA metabolic process and mucus secretion were among the most significantly enriched biological processes.

Table 3 shows the results of pathway topology analysis by Signaling Pathway Impact Analysis (SPIA). SPIA uses the information from a set of differentially expressed genes and their fold changes, as well as pathways topology in order to assess the significance of the pathways in the condition under the study [31]. These two aspects are captured by the probability values: P_NDE_ (probability of obtaining a number of differentially expressed genes on the given pathway at least as large as the observed one), where NDE stands for the number of differentially expressed genes and P_PERT_ which is calculated based on the amount of perturbation measured in each pathway. The two types of evidence, *P_NDE_* and *P_PERT_*, are finally combined into one global probability value, *P_G_*, which is used to rank the pathways and test the research hypothesis that the pathway is significantly perturbed in the condition under the study. A significance threshold of 5% was used on the FDR corrected p-values (pGFDR) in order to detect pathway significance. The Kyoto Encyclopedia of Genes and Genomes (KEGG) pathway ID enriched in each group are shown in Table 3. Based on this analysis, we found the KEGG pathways common to both males and females (Table 3) were; p53 signaling, cytokine-cytokine receptor interaction, transcriptional misregulation in cancer, mitogen-activated protein kinase (MAPK) signaling, chemokine signaling and toll-like receptor signaling. In male mice, the pathways of dilated cardiomyopathy and NF kappa B signaling were significant based on the DEGs. In female mice, alcoholism, systemic lupus erythematosus (SLE) and transcriptional misregulation in cancer were the significant pathways.

**Table 3:** Signaling Pathway Impact Analysis of DEGs.

Table 3a: DEGs exclusive to females in response to oxygen					
Pathway name (KEGG)	Pathway ID	Size	NDE	pGFDR	Status
*Systemic lupus erythematosus*	05322	124	23	8.46e-18	Activated
*Alcoholism*	05034	175	22	2.15e-13	Activated
*Transcriptional misregulation in cancer*	05202	171	12	0.0004	Inhibited

Table 3b: DEGs exclusive to males in response to oxygen					
Pathway name (KEGG)	Pathway ID	Size	NDE	pGFDR	Status
*Dilated cardiomyopathy*	05414	88	5	0.0460	Activated
*NF-kappa B signaling pathway*	04064	89	6	0.0460	Inhibited

Table 3c: DEGs common to both sexes in response to oxygen					
Pathway name (KEGG)	Pathway ID	Size	NDE	pGFDR	Status
*Rheumatoid arthritis*	05323	80	19	0.0006	Activated
*HTLV-I infection*	05166	275	40	0.0020	Inhibited
*Cytokine-cytokine receptor interaction*	04060	239	34	0.0020	Activated
*p53 signaling pathway*	04115	67	14	0.0020	Inhibited
*Transcriptional misregulation in cancer*	05202	171	30	0.0020	Inhibited
*RNA transport*	03013	156	27	0.0038	Activated
*Focal adhesion*	04510	199	30	0.0161	Activated
*Osteoclast differentiation*	04380	117	20	0.0177	Inhibited
*MAPK signaling pathway*	04010	260	36	0.0177	Activated
*Epstein-Barr virus infection*	05169	204	26	0.0218	Inhibited
*Small cell lung cancer*	05222	83	12	0.0218	Activated
*Chemokine signaling pathway*	04062	177	25	0.0218	Activated
*Axon guidance*	04360	129	20	0.0302	Inhibited
*Toll-like receptor signaling pathway*	04620	100	17	0.0474	Activated

*Notes. The KEGG IDs of the pathways are specified. NDE: Number of differentially expressed genes in the pathway; pGFDR: False discovery rate corrected p value.*

### Real time qPCR validation

**Figure 5:**
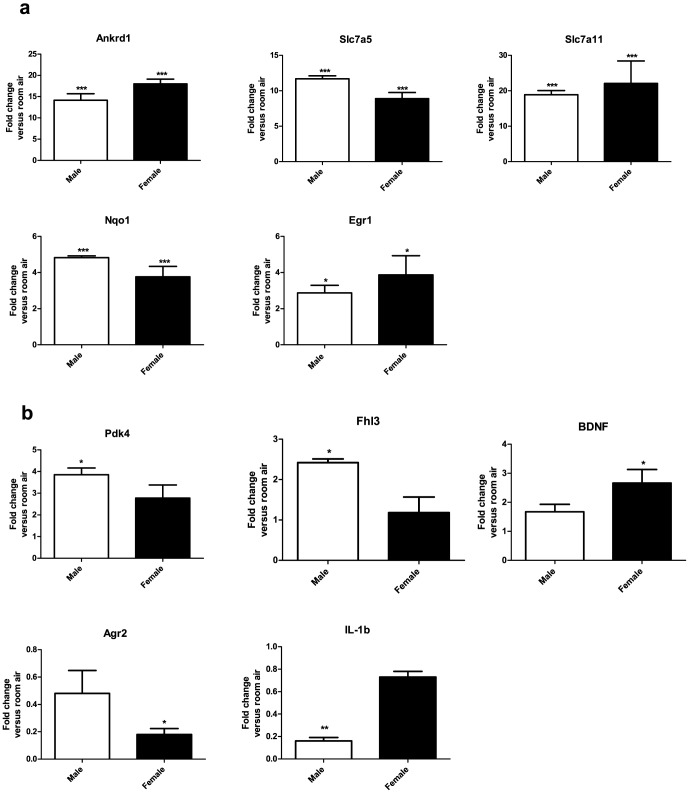
Real time RT-PCR analysis of mRNA from the lungs of male and female mice exposed to room air or hyperoxia for 48 h. Values are means ± SEM from n = 3 groups. Each group consisted of pooled RNA from four animals. Figure 5a: Real time RT-PCR analysis of differentially expressed genes common to both male and female mice. Significant upregulation over room air levels are indicated by * p<0.05 and *** p<0.001(one-way ANOVA). Figure 5b: Real time RT-PCR analysis of genes showing sex-specific changes. Fold change over room air levels are represented on the y-axis. Significant up or downregulation over room air levels are indicated by *p<0.05 and **p<0.01 (one-way ANOVA).

**Figure 6:**
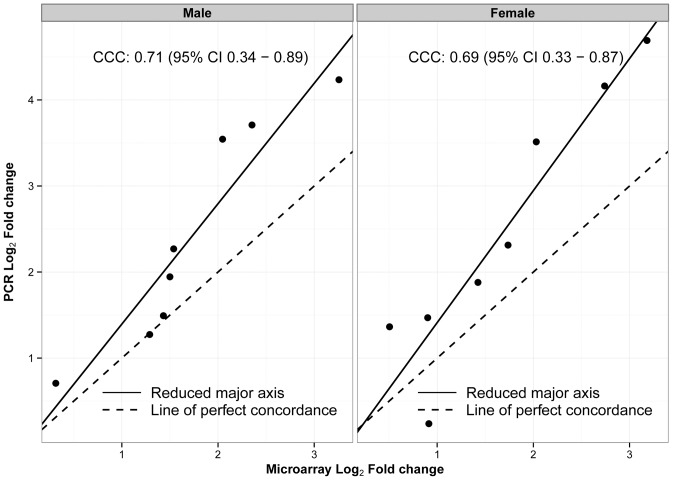
Concordance correlation coefficient (CCC) between qPCR and microarray results. Used to measure the agreement between the log_2_ FC results from qRT-PCR and microarray in both males and females. The dashed line represents the line of perfect concordance. The solid line represents the reduced major axis.

Microarray results were verified by qPCR. Usually, validation of differential expression of genes is performed on a gene to gene basis. The disadvantage of following this approach is that it is not possible to generalize validation results to the remaining majority of non-validated genes or to evaluate the overall quality of these studies. Miron *et al.* [32] showed that the common method of selecting only the most differentially expressed genes for validation generally fails as a global validation strategy and proposed random-stratified sampling as a better gene selection method. Therefore, we selected 5 genes (*Ankrd1*, *Slc7a5*, *Slc7a11*, *Egr1* and *Nqo1*) from the pool of DEGs in a random-stratified manner based on their fold change as described in materials and methods. In addition, we also selected five additional genes from the pool of genes showing sex-specific differential expression in the microarray experiment. Figure 5 shows the PCR results for DEGs common to both males and females (5a) and the changes in transcript levels of genes showing sex-specific differences (5b) in expression after hyperoxia exposure. Table 4 shows the comparison of results between microarray and PCR for the selected genes. To better estimate the global validation of the microarray experiment with the PCR, we calculated the concordance correlation coefficient (CCC), which combines the accuracy and precision coefficients in one index. This is depicted in Figure 6. All the genes tested in males and females by microarray and PCR are represented as dots in this graph. It is used to determine how far the observed data deviate from the line of perfect concordance. CCC increases in value as a function of the nearness of the data's reduced major axis to the line or perfect concordance (the accuracy of the data) and of the tightness of the data about its reduced major axis (the precision of the data). We achieved a CCC of 0.71 for males and 0.69 for females, which shows good validity of our microarray experiment findings [32].

**Table 4:** Fold change for the selected genes in the microarray and qPCR experiment.

Affymetrix ID	Gene Symbol	Gene Name	Group	Microarray		PCR	
				M	F	M	F
10498024	Slc7a11	Solute carrier family 7 (cationic amino acid transporter, y+ system), member 11	C	9.513	9.063	18.83	25.83
10467191	Ankrd1	Ankyrin repeat domain 1	C	5.098	6.68	13.08	17.91
10582275	Slc7a5	Solute carrier family 7 (cationic amino acid transporter, y+ system), member 5	C	4.141	4.084	11.67	11.42
10454782	Egr1	Early growth response 1	C	2.694	3.340	2.82	4.97
10581538	Nqo1	NAD(P)H dehydrogenase, quinone 1	C	2.908	2.676	4.82	3.68
10474399	BDNF	Brain derived neurotrophic factor	F		1.424	1.63	2.57
10543017	Pdk4	Pyruvate dehydrogenase kinase, isozyme 4	M	2.83		3.85	2.77
10487597	IL1b	Interleukin 1 beta	M	−2.55		0.16	0.73
10395365	Agr2	Anterior gradient 2	F		−2.64	0.48	0.18

*Notes:* ***C*** *: Common to both male and female animals;* ***F*** *: In females only;* ***M*** *: In males only.*

### Immunohistochemistry and western blot

**Figure 7:**
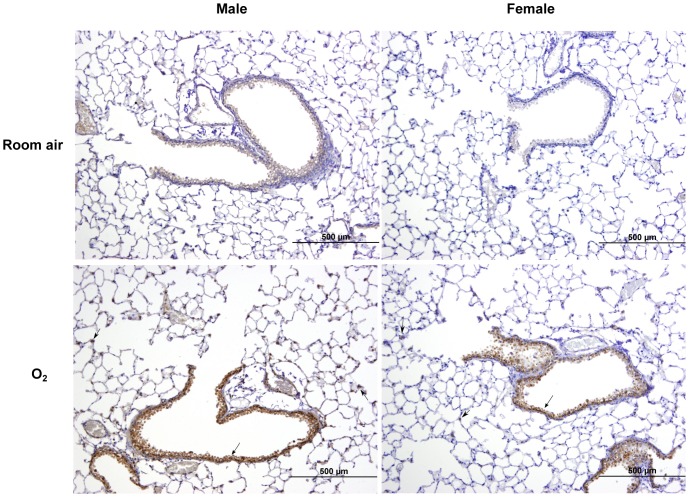
Increased Slc7a11 expression in the lungs (male or female) following hyperoxia exposure. Representative sample of immunohistochemical staining for *Slc7a11* in the lungs of male or female mice at room air and after exposure to hyperoxia for 48 h (n = 5 animals/group).

**Figure 8:**
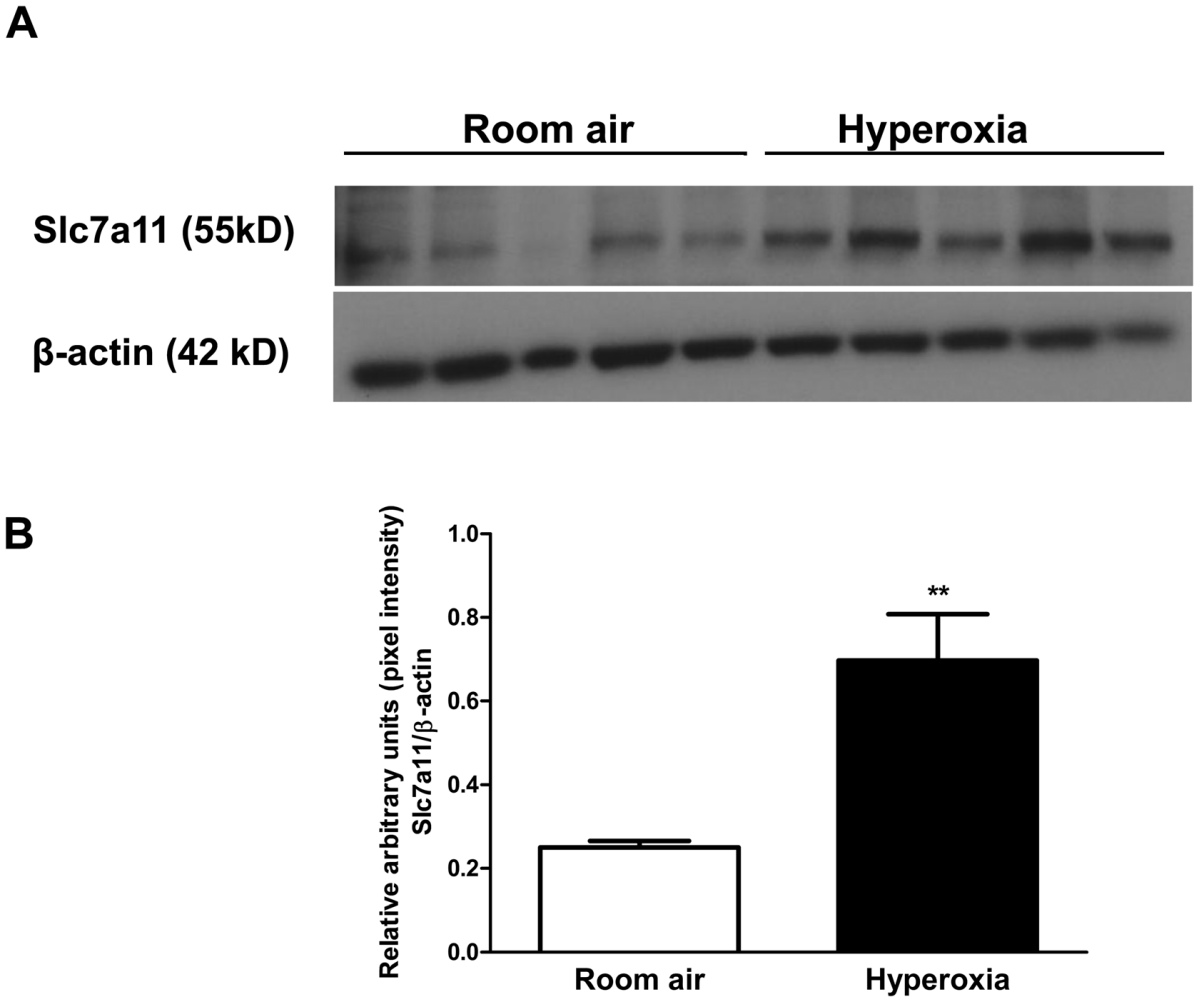
Increased Slc7a11 expression in the lungs (male) following hyperoxia exposure. **8A:** Western blot assay for Slc7a11 expression in lungs following hyperoxia exposure. Twenty µg of whole lung protein from male animals at room air and exposed to 48 h of hyperoxia was subjected to western blotting using antibodies against Slc7a11. Under each sample lane is the corresponding β-actin blot to assess for protein loading. **8B:** Densitometry analysis of pulmonary Slc7a11 immunoblots at room air and after 48 h of hyperoxia exposure. Significant differences from room air controls are indicated by **, p<0.01.

For validation at the protein level we performed immunohistochemistry for one of the DEGs (*Slc7a11*) in the hyperoxia group, which was common to both males and females. Compared to the room air controls, after 48 h of hyperoxia exposure, intense positive staining is seen in the lung sections in the peribronchiolar epithelial and type II alveolar epithelial cells (Figure 7) similar to the results seen in the microarray and the PCR results. Figure 8A shows Slc7a11 protein expression assessed by western blot assay in whole lung protein from male mice, at room air and after 48 h hyperoxia exposure. There is increased expression (8B; p<0.01) of Slc7a11 after hyperoxia exposure compared to room air controls. The expression pattern was similar in females (results not shown).

## Discussion

The major new findings of this study are the identification of new candidate genes of interest and the sex-specific transcriptomic changes in hyperoxic lung injury. We also identified pathways that are differentially regulated in a sex-specific manner. These findings could in part explain the differences in susceptibility between males and females in diseases such as ARDS and BPD. The hyperoxic lung injury model is a type of oxidant stress to the lung and the findings from this study could explain the sex-specific differences in the pathophysiology of many other diseases, in which increased oxidative stress plays a role such as pulmonary fibrosis, asthma and COPD [36]. While transcriptomic differences have been described in hyperoxic lung injury [37] sex-specific changes in the transcriptome following hyperoxic lung injury have not been not been well described in the past studies.

Using microarrays to examine differential gene expression in the lungs after hyperoxia exposure for 48 hours, we identified genes that are differentially expressed unique to each of the sexes as well as those that are common to both the sexes. Of those genes that were differentially expressed following hyperoxia exposure, 1882 were common to both males and females; there were 585 genes unique to males and 327 unique to females. We selected the 48 h time point, as major differences in lung injury by histopathological analysis and markers of inflammation were found at 72 h with most animals dying between 60–90 h of exposure [6]. This study, therefore, focuses on the initiation phase of hyperoxic lung injury when cellular activation instead of cellular injury is taking place [5].

The study reveals potential candidate genes that could explain the sex-specific differences in the pathophysiology of hyperoxic lung injury. Of particular note are the genes *Pdk4, Fhl3, BDNF, Agr2 and CCl17*. *Pdk4* (pyruvate dehydrogenase lipoamide kinase 4), was upregulated in males after hyperoxia exposure. *Pdk4* is an inhibitory kinase, which phosphorylates and inhibits the activity of pyruvate dehydrogenase. The role of this gene in hyperoxic lung injury has not been tested. Grassian *et al* (2011) reported that *Pdk4* overexpression decreased de novo lipogenesis and cell proliferation. Hyperoxia exposure is known to decrease cell proliferation in pulmonary epithelial cell lines when exposed to hyperoxia [38], and *Pdk4* could have a potential role in this effect. Another candidate gene, *Agr2* was downregulated in female mice after hyperoxia exposure. *Agr2* inhibits p53 [39] and hyperoxia is known to induce p53 expression [40]. The downregulation of *Agr2* in female could in part explain this phenomenon in female mice. *Fhl3* (The four-and-a-half LIM domain 3) gene was upregulated in males after hyperoxia exposure. The four-and-a-half LIM domain (FHL) proteins have a highly conserved double zinc finger motif and have been implicated in transcriptional regulation [41] and suppression of tumor growth [42]. *Fhl3* has been shown to inhibit the transactivation of hypoxia-inducible factor 1 (*HIF-1*) [43]. *HIF-1* in turn has been shown to be downregulated in neonatal mice exposed to hyperoxia [44], [45]. *Fhl3* could also have effects on cell cycle progression and apoptosis. It has been shown to cause cell-cycle arrest through upregulation of p21 and induce caspase-3 mediated apoptosis in glioma cells [46]. *BDNF* (Brain derived neurotrophic factor) was upregulated in females following hyperoxia exposure. Neurotrophins like BDNF have been implicated in many lung diseases [47]. *BDNF* was upregulated in the lung specifically in the peribronchial smooth muscles in 5- day old rat pups exposed to >95% oxygen for 7 days [48]. *Ccl17* was upregulated in females after hyperoxia exposure. This gene is one of several Cys-Cys (CC) cytokine genes. The cytokine encoded by this gene displays chemotactic activity for T lymphocytes, but not monocytes or granulocytes. In a study by Bhattacharya *et al* [49] in human lung samples in patients with BPD, *CCl17* was downregulated.

Pathway analysis based on sex-specific gene expression patterns showed sex-specific differences in enrichment of biological processes (Figure 4 and Table 3). The MAP kinase pathway is activated under hyperoxic conditions [50]–[52]. At the 48 h time point, in our study, the MAP kinase pathway was inhibited in males but activated in females. Interestingly, *IL-1β* was found to be downregulated in males at the 48 h time point and not in females and this could explain the differential regulation of this pathway in males. Similarly, the NFκ-B pathway was inhibited in males by pathway topology analysis. NFκ-B also plays an important role in hyperoxic lung injury [53]–[55]. The pathway is activated in hyperoxic conditions but this may be a part of a survival mechanism to escape cell death [56]–[58]. Enhanced NF-κB protected the lung from acute hyperoxic injury in neonatal mice via inhibition of apoptosis (Yang *et al.*, 2004). Specifically, *IL-1β* was downregulated and *Iκbα* was upregulated (data not shown) both of which could play a role in the inhibition of this pathway. Also, higher levels of oxidative stress can lead to inhibition of NFκ-B rather than its activation through other mechanisms [55].

In addition to the genes, which showed sex-specific changes in their response to hyperoxia exposure, we identified important differentially expressed genes common to both sexes. Gene expression pattern in the lung after exposure to hyperoxia has been reported previously in adult and neonatal mice. In the study by Perkowski *et al* in 2003 [59] using a total of five mice, *p21, metallothionein and cysteine-rich protein 61* were the top three upregulated genes in 8–10 week old female mice after hyperoxia exposure for 48 h. We also found these genes to be upregulated both in male and female mice in our study at this time point. We also found similar upregulation in *elastin, galectin 3, glutathione reductase, heme oxygenase-1, p53 and connective tissue growth factor*. *connective tissue growth factor*.

*Slc7a11* (solute carrier family (anionic amino acid transporter light chain Xc-system), Member 11) was upregulated in both male and female mice in our study and we validated this at the protein level using immunohistochemistry and western blot (Figure 7 and 8). To our knowledge, this is the first documentation of induction of this gene and protein in a hyperoxic lung injury model in adult mice. This gene codes the protein, xCT, which is upregulated after exposure to hyperoxia [60]. xCT is regulated in part by *Nrf2*, which has an important protective role in hyperoxia induced ALI [61]. Howden *et al* [62] exposed adult mice to 100% oxygen for 96 hours in different mouse strains to study the cardiopulmonary response and identify candidate susceptibility genes in hyperoxic lung injury. *Slc7a11* emerged as one of the candidate genes by linkage analysis. *Slc7a11* was also upregulated in neonatal mice following hyperoxia exposure [37]. The protective role of xCT against oxidative stress in the lung was also shown in a model of paraquat induced oxidative stress. xCT deficient mice had decreased survival, increased injury and inflammation. It was upregulated in lungs exposed to oxidative stress and contributed to the maintenance of glutathione levels under these conditions [63].

*Nqo1* (NAD(P)H:quinone oxidoreductase 1) is a phase II antioxidant enzyme that has been shown to be induced in murine lung in response to hyperoxia by *Nrf2* to protect against lung injury [37] was upregulated following 48 h of hyperoxia exposure in our study. Single nucleotide polymorphisms in this gene have been linked to susceptibility to ALI [64]. *Serpine-1 or plasminogen activator inhibitor (PAI)-1* was also upregulated in our study. This gene has been shown to play a major deleterious role in hyperoxic lung injury [65]. *PAI-1* upregulation impairs fibrinolytic activity in the alveoli and leads to fibrin accumulation.

We have reported previously on sex-specific differences in hyperoxic lung injury and the possible protective role of cytochrome P450 (CYP)1A1. We did not find this gene to be significantly differentially regulated at the 48 h time point in this study. In our experiments *Cyp1a1* shows the most upregulation at the 24 h time point (data not shown), which could explain its absence in the list of DEGs at the 48 h time point.

Although the array data provides a comprehensive overview of gene expression in the lung after hyperoxia, our study has its limitations. First, array data provides information on gene expression levels. Changes in gene expression levels may not necessarily reflect actual protein levels. Also, important functional changes induced by post-translational modifications cannot be identified. In this study, we examined whole lung tissue gene expression that includes endothelial cells, epithelial cells, interstitial cells, as well as circulating or adherent hematopoietic cells. Hence, most of the genes on the array are likely to be expressed by many cell types. In this investigation, we did not time our experiments with the estrous cycle in female mice, which could have led to some of the variability in our findings. The data is from 12 female adult mice whose lung RNA was pooled in this study. However, there is emerging data that mammalian cells differ intrinsically based on the sex and also respond differently to stressors irrespective of the past or current concentrations of sex hormones [66].

In conclusion, we present the changes in the mouse pulmonary transcriptome after hyperoxia exposure at the 48 h time point and identified new potential candidate genes of interest. Furthermore, our results support the hypothesis that there is sex-specific modulation of gene expression and biological processes in the lung under hyperoxic conditions, which may contribute to sex-based differences in hyperoxic lung injury. It is increasingly appreciated that gender differences impact disease presentation and clinical trajectory of patients with ALI. While hormonal factors and differences in lung development are described as contributing factors, comprehensive evaluation of the transcriptomic profile as in this study provides a substantial opportunity to advance the understanding of sex-specific differences in the pathophysiology of hyperoxic lung injury, and could lead to insights about sex-related individualized therapy.
